# A Combinatorial Approach to Detect Coevolved Amino Acid Networks in Protein Families of Variable Divergence

**DOI:** 10.1371/journal.pcbi.1000488

**Published:** 2009-09-04

**Authors:** Julie Baussand, Alessandra Carbone

**Affiliations:** 1 Génomique Analytique, Université Pierre et Marie Curie, Paris, France; 2 Génomique des Microorganismes, CNRS, Paris, France; Fox Chase Cancer Center, United States of America

## Abstract

Communication between distant sites often defines the biological role of a protein: amino acid long-range interactions are as important in binding specificity, allosteric regulation and conformational change as residues directly contacting the substrate. The maintaining of functional and structural coupling of long-range interacting residues requires coevolution of these residues. Networks of interaction between coevolved residues can be reconstructed, and from the networks, one can possibly derive insights into functional mechanisms for the protein family. We propose a combinatorial method for mapping conserved networks of amino acid interactions in a protein which is based on the analysis of a set of aligned sequences, the associated distance tree and the combinatorics of its subtrees. The degree of coevolution of all pairs of coevolved residues is identified numerically, and networks are reconstructed with a dedicated clustering algorithm. The method drops the constraints on high sequence divergence limiting the range of applicability of the statistical approaches previously proposed. We apply the method to four protein families where we show an accurate detection of functional networks and the possibility to treat sets of protein sequences of variable divergence.

## Author Summary

Fine analyses of families of protein sequences reveal the existence of networks of coevolved amino acids. These networks are clusters of residues often entering in physical contact one with the other, and they relate residues which are located far apart on the three dimensional structure. Coevolved residues often play a major biological role in the protein, and the nature of their interactions might be multiple, spanning among binding specificity, allosteric regulation and conformational change of the protein. By carefully tracing the way residues evolved within the phylogenetic tree of sequences of a protein family, the Maximal SubTree Method captures the transition along the time scale evolution of a conserved position to a coevolved position, and provides a numerical evaluation of the degree of coevolution of pairs of coevolved residues in a protein. This combinatorial approach drops the constraints on high sequence divergence limiting the range of applicability of the statistical approaches previously proposed, and it can be applied with high accuracy to families of protein sequences with variable divergence.

## Introduction

The function and mechanical properties of a protein demand strong evolutionary pressures along evolution which are directed on the one hand, to conserve residues involved in catalytic sites and in interactions with amino acids of other proteins [1]–[4], and on the other hand, to mutually conserve residues involved in networks of interacting residues lying within the protein itself [5],[6]. Studies of many protein complexes indicate that long-range interactions of amino acids are as important for the functional mechanisms of the protein (binding specificity, allosteric regulation, conformational change) as residues directly contacting the substrate. A theoretical understanding of these experimental observations leading to rigorous definitions of conservation and coevolution would provide a framework for the development of methods to predict and analyze groups of conserved and coevolved residues. Two positions in a protein sequence are *conserved* under ”independent” events and are *coevolved* under “correlated” events, where an event is some evolutionary pressure imposed for functional or structural reasons. To measure in a precise manner different degrees of coevolution (where conservation is identified to have maximal degree) is central to the understanding of coevolution. To tackle this problem means to propose a method to quantitatively measure coevolution of positions in aligned sequences and to identify clusters of positions following similar patterns of coevolution.

Several methods investigating evolutionary constraints in proteins via the analysis of correlated substitutions of amino acids have been proposed. Sequence-based statistical methods analyze covariations between positions of aligned sequences by using correlation coefficients [7],[8], mutual information [9]–[11], and deviance between marginal and conditional distributions to estimate the thermodynamic coupling between residues [5],[12],[13]. Phylogenetic information has been coupled to the statistical approach in [14], and it is used to better treat sequences displaying the same level of covariation, being this latter generated by either a few independent substitutions in early ancestors or correlated changes along multiple lineages [15],[16]. A non-equilibrium molecular dynamics simulation method has also been proposed which measures the anisotropic thermal diffusion (ATD) of kinetic energy originating from a specific residue. It extracts the signaling pathway in which the residue is involved in the protein [17]. Finally, those residue positions which are determinant for the highest residue interconnectivity within a protein family have been shown to be crucial for maintaining short paths in network communication and to mediate signaling [18],[19]. Some of these residue positions are also found in networks of statistically coupled residues predicted by Suel & Ranganathan [5].

We propose a sequence-based combinatorial alternative to statistical approaches for the detection of functionally important coevolved residue networks using phylogenetic information. This combinatorial approach is based on the analysis of a set of aligned sequences, on the associated distance tree and on the combinatorics of its subtrees and does not need structural data nor the knowledge of functional residues as the ATD method. The first stage of the method selects conserved positions based on the scattering of residues (within the position) in the tree. For this, a novel notion of rank for an alignment position in a multiple sequence alignment is used. It is purely based on information extracted from the distance tree, and it is defined to be the number of Maximal SubTrees (MST) observed at the position, where a MST is the largest subtree conserving a residue at the given position. In the second stage of the method, all pairs of selected conserved positions are evaluated accordingly to the distribution of their residues in the tree. Namely, for each selected position, we parse the distance tree and apply numerical criteria to score coevolution between pairs of residues conserved on subtrees and identify positions with similar residue distribution.

We apply the method to the haemoglobin and serine protease families, which have been previously studied by Suel & Ranganathan with the Statistical Coupling Analysis (SCA) approach [5],[12]. For this, we use the same alignments of highly divergent sequences which satisfy stastitical constraints. The MST method captures with the same accuracy the networks detected by SCA and it predicts some new coevolved positions missed by SCA because of the number of aligned sequences and of sequence divergence which are required to be both high by the statistical approach. In general, these constraints limit the domain of applicability of SCA to well-described families. We successfully apply the MST approach to the leucine dehydrogenase and PDZ domain families and base the analysis on sequences selected with PSI-BLAST, with no divergence constraints and only one reference sequence. Mechanical and functional networks have been detected for both families.

## Methods

### Rank of a position in a sequence alignment

**Figure 1:**
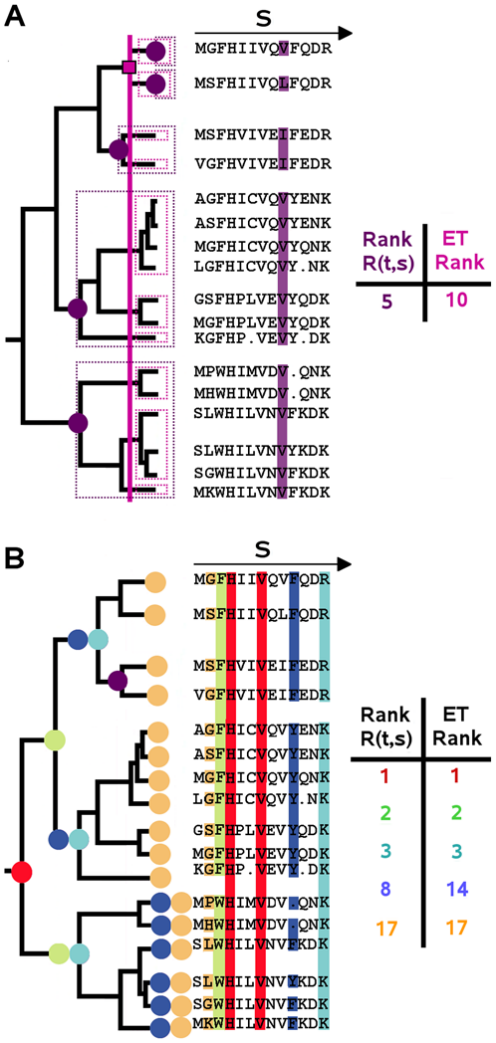
MSTs and ranks illustrated in a sequence alignment and associated distance tree T. A. Analysis of conservation at position s = 9 in the sequence alignment, MST rank 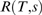 and ET rank (as defined in [20]). The 5 MSTs conserving residues at position 9 are delimited by purple dotted lines and their roots are represented by purple circles. The 10 subtrees identified by the ET approach are delimited by pink dotted lines and the node determining the rank of conservation of the 9th position is indicated by a pink square. B. Analysis of 6 different alignment positions marked with distinguished colors in the alignment and in the tree. The rank 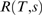, its corresponding ET rank and the roots of MSTs decomposing *T* with respect to position *s* are colored the same way.

The rank of a position *s* in a tree *T* corresponds to the number of MSTs decomposing *T* at position *s*, where a MST is the largest subtree conserving a same residue (see Figure 1A).

Let *T* be a tree associated to some aligned set of sequences, 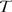 be its nodes, 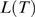 be its leaves each labeled with an aligned sequence, 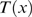 be the subtree of *T* rooted at 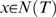, and 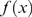 be the father node of 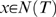, if it exists. If *S* is the length of the alignment, then we distinguish *S* different positions. Let 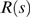 be the set of residues belonging to the aligned sequences at position 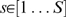, and 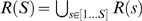. The function 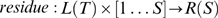 associates to a leaf *l* of *T* and to a position *s* the residue *r* corresponding to the *s*-th position in the aligned sequence labeling the leaf *l*, with 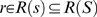.

A subtree 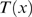 is *conserved at position s* if 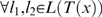, 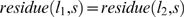. By convention, gaps are considered to be different residues, and if both 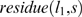 and 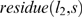 are gaps then 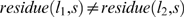. A subtree 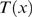 is *maximal at position s* if 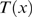 is conserved at position *s* and, if 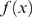 exists then 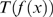 is not conserved at *s*.

A *rank of a position s in T* is defined as
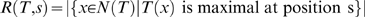
with 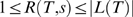 (see example in Figure 1).

This new definition of rank differs from the one initially used in the Evolutionary Trace method (ET) [20] which corresponds to the minimal distance from the root determining subtrees that conserve a same residue. The ET rank is easily affected by erroneous alignments and incorrect tree constructions as shown in Figure 1A, where the ET rank is required to be very low even though the residue *V* is conserved in almost all sequences at position 9. It also differs from definitions which combine tree structure information with information content of aligned sequences [21] or from definitions combining tree structure information with physico-chemical properties of the residues [22]. A rank 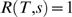 means that *T* is maximal at position *s*, that is, *s* is completely conserved (see red positions in Figure 1B), and a rank 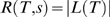 means that each leaf in *T* at position *s* is a MST, that is, each pair of neighboring leaves in the tree is associated to different residues at position *s* (see the orange position in Figure 1B). Intuitively, positions with small (big) rank have undergone strong (weak) evolutionary pressure.

### Selection of seed positions

To identify networks of coevolved residues, we work under the hypothesis that coevolved positions are “enough conserved”. For this, we shall select a group of starting positions, called *seeds*, which display a sufficiently high conservation level.

#### Conserved positions and stability

We consider any gap occurrence as representing a different residue. This implies that highly gapped positions will be ranked high. We could have chosen to consider gaps as a specific residue and in this case highly gapped positions would have been ranked the lower. The rank distribution and the mean rank calculated over all alignment positions turn out to be strictly dependent on the definition one chooses (see Figure S1).

Let 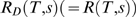 be the rank of position *s* in *T* and 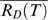 be the mean rank calculated over all alignment positions in *T*, when aligned gaps are considered as *different* (*D*) residues. 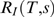 and 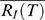 denote the rank of position *s* in tree *T* and the mean rank calculated over all alignment positions in *T* when aligned gaps are considered as an *identical* (*I*) residue. A *stable* position *s* in *T* is such that 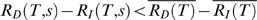, that is a position whose rank is not much affected by gaps.

Let 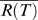 be the mean rank calculated over all stable positions in *T*. A position *s* in *T* is *conserved* if 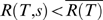. The intuition here is to identify (and select for the analysis) as conserved those positions exhibiting a stronger signal of conservation than the average.

Since simple variations in sequences can lead to different tree decompositions of *T* in MSTs, position ranking and mean ranks, we want to check the robustness of the conservation for a position over a number of landmark points on *T*, called *checkpoint nodes*. Below, we formally describe how to select checkpoint nodes in *T* accordingly to sequence divergence, and how to evaluate *persistency* of conservation of a position in all subtrees of *T* rooted at checkpoint nodes.

#### Checkpoint nodes

Checkpoint nodes are selected in *T* going from the leaves of the tree up to the root. The first checkpoint nodes are roots of the smallest subtrees of *T* whose corresponding sequences present at least 60% of mutated positions, that is 60% of positions in the aligned sequences have rank>1. The minimal sequence divergence defining the lowest checkpoint nodes is supported by the observation that generally, important functional divergence in homologous sequences appears under the threshold of 40% sequence identity [23],[24] and that the first three digits of an EC number can be transferred with confidence between proteins presenting at least 40% sequence identity [25]. The intuition is that conserved positions detected in sequences under this threshold are supposed to undergo strong evolutionary pressure and be functionally relevant.

Checkpoint nodes with higher sequence divergence are defined inductively to be nodes *x* in *T* which present at least 10% of mutated positions more than the checkpoint node *y* with highest divergence lying below *x*. A minimal increase of 10% in sequence divergence in *x* is asked between successive checkpoint nodes in order to favor diversity of subtrees in which positional conservation is evaluated. Jumps on 10% mutated positions are a way to discretize the tree by avoiding an evaluation on all its nodes that could be affected by phylogenetic effects (certain branches could be more populated with very similar sequences) leading to an overestimation of conservation signals.

Finally, a node in the tree that reached 90% of mutated positions as well as its immediate children, is considered to be a checkpoint node.

#### Persistent conservation of a residue

**Figure 2:**
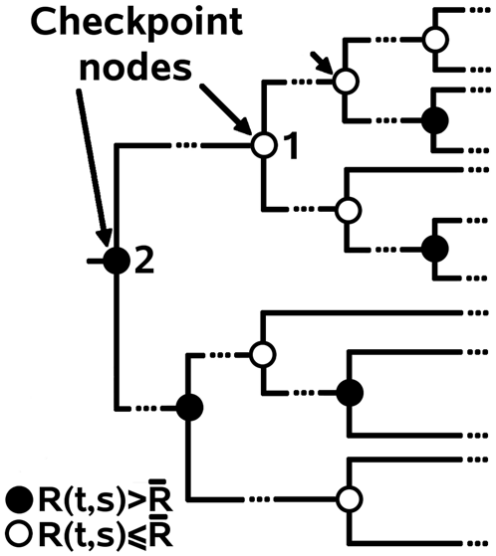
Checkpoint nodes. Distance tree with checkpoint nodes colored differently depending on the conserved status of a position *s* in the subtree *t* rooted at the checkpoint node: grey if *s* is not conserved, and white if *s* is conserved. All nodes in the tree which are not checkpoint nodes, and that are possibly located between two checkpoint nodes, are not indicated. At the white checkpoint node 1, the longest monochromatic path starting at 1 and going towards the leaves has length 3. This asks for 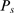 to be incremented by 3. At the grey checkpoint node 2, the longest monochromatic path starting at 2 and going towards the leaves has length 2, and this asks for 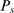 to be decremented by 2.

At each checkpoint node *x*, the mean rank 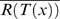 calculated over all stable positions in 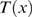, is compared to the rank 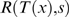 at each position *s*. The *persistency of conservation of a position s* is identified by a persistency score 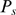 modified at each checkpoint node in the tree accordingly to the conserved status of the alignment position *s* within the subtree rooted at this node. If a position *s* is conserved at checkpoint node *x* (i.e. 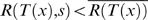), 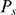 is incremented of a weight 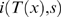 corresponding to the maximal number of consecutive checkpoint nodes encountered on a path of the tree 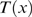 from *x* down to some leaf. If a position *s* is not conserved (i.e. 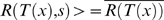), 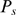 is decremented of a weight 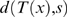 corresponding to the maximal number of consecutive checkpoint nodes where *s* is not conserved encountered on a path of the tree 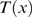 from *x* (included) down to some leaf (see Figure 2).

At the root of *T*, 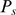 measures the stability of conservation for a position *s* in *T*. Positions conserved in all subtrees rooted at checkpoint nodes have a positive persistency score 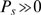 and positions conserved in none of the subtrees rooted at checkpoint nodes have a negative persistency score 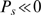. The persistency score of other positions might take a positive or negative value accordingly to the global conservation evaluated at different checkpoint nodes. Positions with a positive persistency score 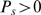 at the root of *T* are considered as *persistently conserved* and they are selected as seed positions for the analysis of coevolving residues.

Notice that not all seed positions are guaranteed to belong to some coevolving network at the end of the analysis. Seeds display some evolutionary pressure and consistent behaviour along the tree, and in this respect they form a set of potential coevolving residues where to restrict the analysis so to reduce the overall computational time coming from the large number of combinatorial residue coupling. Notice, also, that the thresholds defining checkpoint nodes along the distance tree provide a computationally fast manner to avoid phylogenetic effects that might contribute negatively to persistency conservation.

### Combinatorics of MSTs and correspondence scores between pairs of residues

**Figure 3:**
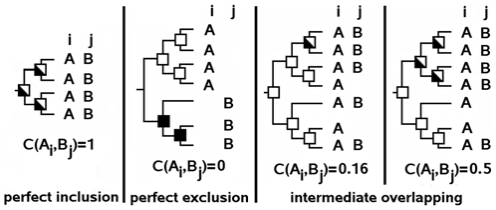
Overlap of MSTs and correspondence scores. Different inner trees specific of residues *A* and *B* at positions *i* and *j* and their corresponding correspondence scores. White squares identify nodes of 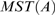 (leaves excluded) and black squares identify nodes of 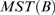. White and black squares identify common nodes between 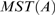 and 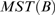. The first two trees illustrate perfect inclusion and exclusion. The last two trees illustrate intermediate cases where the number of sequences with residues *A* and *B* are equal but correspondence scores are different due to a different distribution of sequences in the tree.

**Figure 4:**
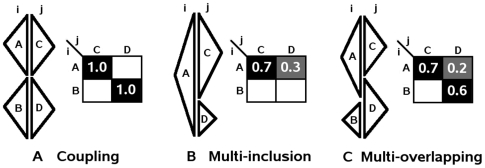
Correspondence matrices and matrix patterns. Two positions 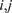 are represented by two residues each. MSTs for these residues are represented by triangles with the associated residue indicated in the center. A. Coupling pattern with an identity correspondence matrix. B. Multi-inclusion pattern where a single residue at position *i* is associated to several residues at *j*. C. Multi-overlapping pattern where several residues at *i* are associated to several residues at *j*.

To evaluate the coevolution of a pair of seed positions, we proceed in two steps. First, we analyze the combinatorics of MSTs associated to a pair of residues at these seed positions and construct a correspondence matrix summarizing the degree of coevolution between all pairs of residues occurring at the seed positions. In a second step, coevolution scores for pairs of seed positions are inferred from the correspondence matrix. They represent how well MSTs associated to a position mirror MSTs associated to another position compared to what would be expected for ideally coevolved positions (see “perfect inclusion” in Figure 3 and Figure 4A).

#### Correspondence matrix construction

**Figure 5:**
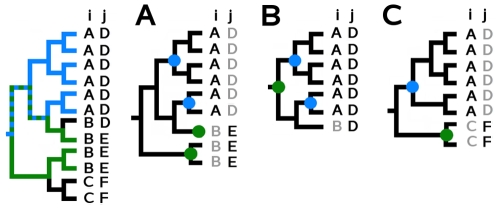
Inner trees. A tree *T* (left) and “inner” trees 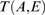 specific of residues *A* and *E* (1), 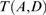 (2), 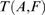 (3) at positions *i* and *j* respectively. Only residues at positions *i* and *j* of aligned sequences are taken into consideration. Branches of *T* labeled with *A* at position *i* and with *E* at position *j* are colored in blue and green respectively and determine the inner tree 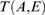 (1). The inner trees 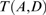 (2) and 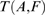 (3) are determined in a similar way. Blue circles in 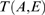, 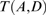 and 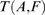 identify roots of MSTs associated to residue *A* at position *i*, and green circles identify roots of MSTs associated to residues *E*, *D* and *F* at position *j*.

Let 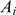 be a residue at seed position *i*. For each pair of residues 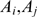 at seed positions 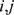, we consider the “inner” tree 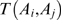 of *T* for which only the leaves of *T* which are labelled by the residue 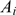 at position *i* or by 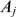 at position *j* are considered (see examples in Figure 5). The inner tree is used to evaluate the overlap of MSTs associated to 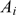 and 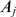. We denote 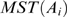 the set of all MSTs associated to a residue 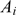 at seed position *i*.

A correspondence score 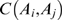 is assigned to each pair of residues 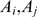 at positions 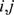:
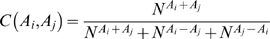
where 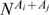 is the number of nodes (leaves excluded) that are common to 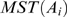 and 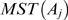, 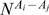 (resp. 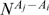) is the number of nodes (leaves excluded) of 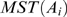 (resp. 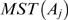) that do not belong to 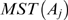 (resp. 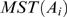). Correspondence scores vary between 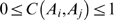 with 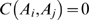 in case of a perfect disjunction of 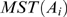 and 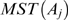, and 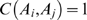 in the case of a perfect inclusion of 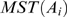 and 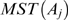 (Figure 3).

Correspondence scores are calculated for each pair of residues 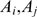 for seed positions 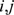 and they are organized in a correspondence matrix 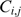 indexed by residues from the most to the least frequent (an arbitrary order is followed for equal frequencies). A row (column) indexed by 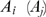 (*A_j_*) contains all correspondence scores obtained by 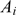 (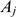) with residues at positions *j* (*i*). The sum of correspondence scores on each line and on each column of matrix 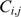 is at most 1.

#### Patterns in a correspondence matrix

Specific patterns might appear in the correspondence matrix accordingly to the combinatorics of the MSTs associated to pairs of residues. The evaluation of a position *i* with itself, for instance, corresponds to the ideal case of coevolution and is characterized by a *perfect inclusion* of MSTs associated to the same residue (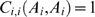) and by a perfect disjunction of MSTs associated to all other residues (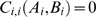). This “perfect” configuration corresponds to an identity matrix. In the case of a pair of independent positions 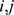, a random overlapping of the MSTs 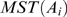 and 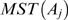 is expected instead.

Patterns in matrices capture three kinds of relations between MSTs associated to pairs of positions:

1. *coupling*: MSTs associated to residues at position *i* mirror MSTs associated to residues at position *j*. This correspondence is represented by an identity correspondence matrix (Figure 4A).
2. *multi-inclusion*: a MST associated to a residue at position *i* (*j*) includes several MSTs associated to different residues at position *j* (*i*). In Figure 4B, residue A obtains its best correspondence score with residue C (since it overlaps mostly with C) but it *lacks specificity* for C since 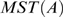 also includes 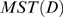. Residues C and D are A specific since they do not overlap with any other MST at position *i*.
3. *multi-overlapping*: MSTs associated to different residues at position *i* overlap with MSTs associated to several residues at position *j*. In Figure 4C, residue A shares residue D with B. The *interference* of MST(D) with 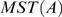 neither *excludes* nor *includes* 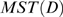 in 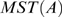.

**Figure 6:**
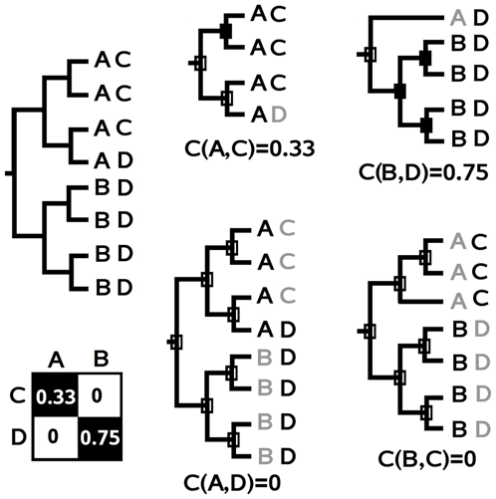
Tree analysis of the residue distribution over two positions and the associated correspondence matrix. Each position is occupied by two residues, with 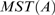, 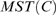 and 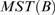, 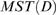 essentially mirroring each other (see tree, top left). Correspondence scores calculated for inner trees (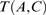 (1), 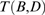 (2), 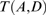 (3) and 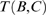 (4)) are reported in the correspondence matrix (bottom left). Within an inner tree defined for a pair of residues, nodes (leaves excluded) conserving both residues are represented with filled black squares, and all others by unfilled squares. In this example, correspondence scores end up to be the ratio between the number of filled squares over the total number of squares (see formal definition in the text).

Coupling describes perfect coevolution between two positions. Since it is unlikely to be observed on real sequence data, the evaluation of coevolution between pairs of positions cannot be reduced to a simple assessment on the presence or absence of a perfect identity matrix. In particular, even for a pair of positions with a good overlap of MSTs, noise in the data caused by a single residue disrupting the maximality of the tree can lead to a diagonal matrix which is not an identity matrix. See Figure 6. Thus, we define a coevolution score between two seed positions by evaluating the “distance” between an ideal identity matrix (coupling) and the actual correspondence matrix which displays less regularity (issued by a possible combination of multi-overlapping and multi-inclusion), for all residues associated to the positions.

### Coevolution score for pairs of seed positions

The *coevolution score of two seed positions* 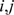 is the sum of two subscores, one evaluating each residue at position *i* accordingly to all residues occupying position *j* and the other evaluating each residue at *j* accordingly to all residues at *i*. For each residue, three multiplicative factors are computed. Intuitively, they numerically describe divergence of the correspondence matrix from the identity matrix, which is expected in the ideal case. In case of perfect coevolution, the three factors will provide no penalties, they equal 1 for all pairs of residues at 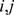, and will make the two subscores equal 1. The more the correspondence matrix diverges from the identity matrix, the more the factors will tend to 0 and will penalize the coevolution score.

#### Maximal correspondence factor

Defined as
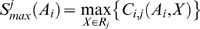
with 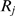 the set of all residues at position *j*, it corresponds to the highest correspondence score obtained for residue 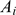 when compared to all residues at position *j*. Note that 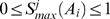. We denote
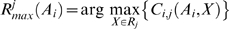
where, by convention, if the maximum of the function is reached on several residues, then 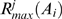 is the most frequent residue among them at position *j*. The maximal correspondence factor penalizes the lack of perfect inclusion among MSTs, which can be due to noise in the data, multi-inclusion or multi-overlapping.

#### Specificity factor

Defined as
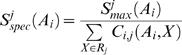
with 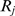 the set of residues at position *j*, it evaluates the specificity of 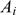 for the residue 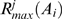. Note that 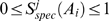. This factor penalizes the lack of specificity which is observed in case of multi-inclusion and multi-overlapping.

#### Interference factor

Defined as
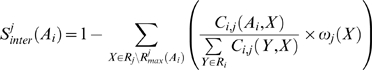
with 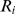 the set of residues at position *i*, 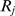 the set of residues at position *j*, 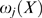 the frequency of residue *X* at position *j*, it evaluates the overlapping between 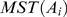 and 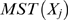 with 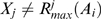. Note that 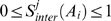. This factor penalizes interference of MSTs at *j* which are not 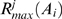 and not completely excluded in MSTs at *i*. Interference is observed in cases of multi-inclusion and multi-overlapping.

Toy examples of 2×2 correspondence matrices are presented in Figure 4. For coupling (Figure 4A), factors for residue A at position *i* are 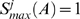, 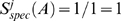 and 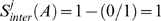, which give 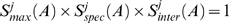. The perfect mirroring of inner trees ensures the correspondence matrix to be the identity matrix.

For multi-inclusion (Figure 4B), factors for residue A at position *i* are 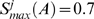, 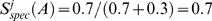 and 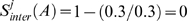, which give 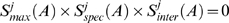. No correlation is observed between positions *i* and *j* since a residue at *j* is associated to two residues at *i* leading to a correspondence matrix far away from an identity matrix. The product of subscores equals 0 and penalizes the configuration. In the more general case of a combination of several residues at *i* and *j* displaying overall a good overlap of their MSTs, local multi-inclusion between pairs of residues might induce a weak penalizing effect on the final score.

For multi-overlapping (Figure 4C), the factors for residue A at position *i* are 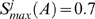, 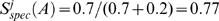 and 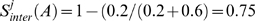, which give 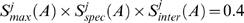. Here the correspondence matrix is closer to the identity matrix and the score is less penalized than in the previous case. However the important multi-overlaps of MST(D) with MST(A), and of MST(D) and MST(A) with MST(C) and MST(D) lead to a rather low product of the subscores, that is 0.4.

#### Coevolution score

The coevolution score 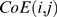 sums up the product of the three factors calculated for each residue in the pair of positions 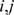 and weights each product accordingly to the frequency of the residue at a given position. We define 
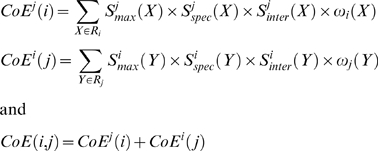
with 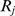 the set of residues at position *j*, 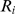 the set of residues at position *i*, 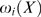 (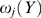) the frequency of residue *X* (*Y*) at position *i* (*j*). Note that 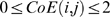 and that 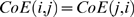.

Notice that pairs of very conserved positions will present a high overlap of their MSTs and obtain high coevolution scores. In the extreme case of two completely conserved positions, the unique MSTs associated to the two positions perfectly mirror each other and lead to a maximal coevolution score of 2.

### A global view of the coevolution analysis

**Figure 7:**
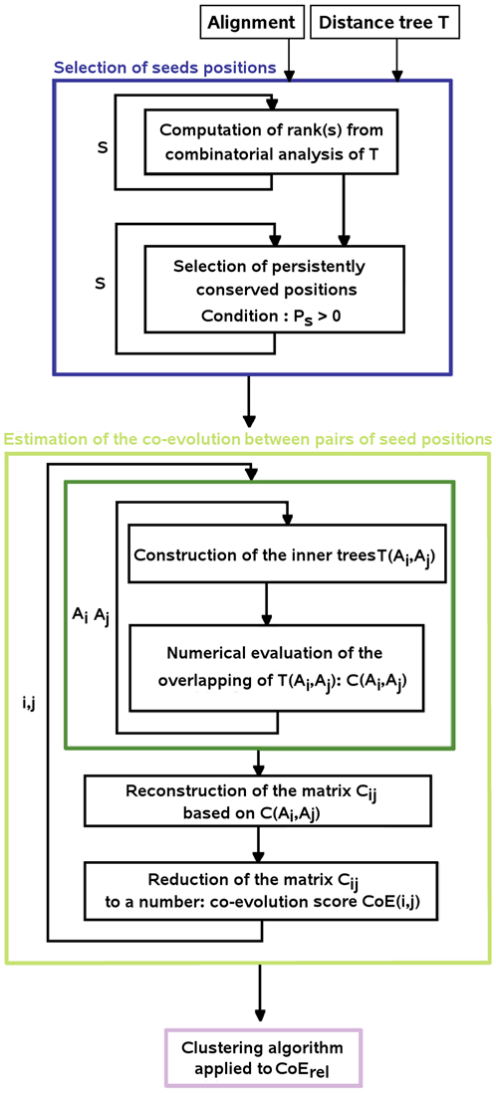
Flowchart of the analysis. The main algorithmic steps of the analysis are represented by three colored boxes. Blue: selection of seed positions; the index *s* runs over all alignment positions. Green: estimation of coevolution between pairs of seed positions; indices 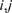 run over seed positions only. Cyan: clustering algorithm; details of the algorithm are presented in Figure 9.

The algorithm is summarised in the flowchart of Figure 7. It takes two inputs, a sequence alignment and a distance tree for the aligned sequences. There are two cut-off values used in the analysis: one concerns sequence variability for checkpoints and the other is expressed in condition 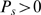. The combination of the two thresholds allows to select seed positions, in the first step of the algorithm (blue box, Figure 7). The full combinatorial analysis of seed positions leading to the detection of coevolving positions does not use any threshold. It is simply based on a combinatorial understanding of how information is distributed on the distance tree and no cut-off value is required (green box, Figure 7).

### Networks reconstruction

#### Domain of variation and relative coevolution score

Each seed position *i* is associated to a *variation domain* defined by the interval 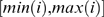, where 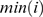 and 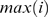 are the lower and higher scores obtained at position *i*, when *i* is combined with other seed positions 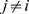. Variation domains of seed positions always overlap with each other, and this is because the coevolution score of a pair 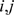 is included in the variation domain of *i* and in the variation domain of *j*.

**Figure 8:**
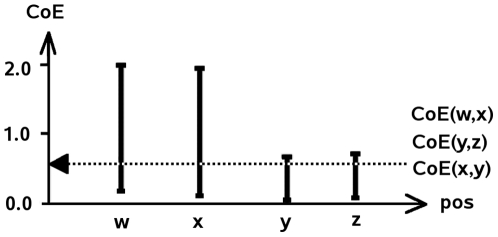
Coevolution score and variation domain. Plot of variation domains for coevolution scores at positions *w*, *x*, *y* and *z* on a toy example. The dotted line shows equal coevolution for pairs of positions 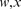, 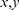 and 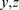. The score of coevolution is low for positions *w* and *x*, but high for positions *y* and *z* with respect to their variation domains. To capture this difference, a normalized score of coevolution is used.

Equal coevolution scores between two pairs of positions do not have necessarily the same meaning with respect to their variation domain, as illustrated in Figure 8. Therefore, it becomes crucial to compare different position pairs only after having normalized their coevolution scores accordingly to variation domains. This is done as follows.

Let 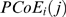 be the relative position of the coevolution score 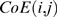 in the variation domain of position *i*

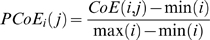
with 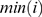 (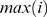) the lower (higher) coevolution score obtained by position *i* with all other seed positions *j*, where 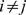.

The *relative coevolution score of a pair of positions* 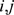 is evaluated accordingly to the coherence of the positions 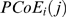 and 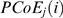 and it is defined by
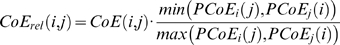



The coevolution score obtained for 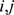 is penalized in the relative coevolution score as much as 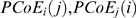 differ. If 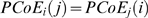 then 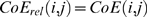.

#### Clustering algorithm

We developed an optimization method that clusters together positions displaying similar best coevolution scores and thus permitting the reconstruction of coevolving residues networks.

The *neighboring set* associated to position *i*, denoted 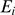, collects the 5 seed positions (including *i*) obtaining the best relative coevolution scores with *i*. The *relative average behavior* of *i* with respect to a position *j* is defined by
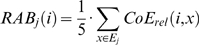



The *difference of relative average behavior* of *i* with respect to *j* is defined as 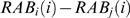.

We denote *P* and 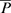, two complementary disjoined sets of seed positions. Sets *P* and 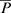 will change along the execution of the algorithm. At the beginning, *P* is composed of exactly one of the seed positions involved in the pair of positions obtaining the higher coevolution score among all possible pairs, and 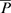 is composed of all other seed positions not included in *P*. *P* is intended to be an ordered set of positions, where the order is imposed by the chronological arrival of a position in the set. The algorithm iteratively selects a position 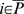 which minimizes the difference of relative behavior with the last position *j* entering the ordered set *P*, such that
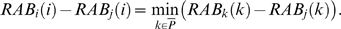



Once selected, position *i* is removed from 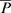 and becomes the last position of *P*. This process is repeated until 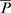 is empty.

**Figure 9:**
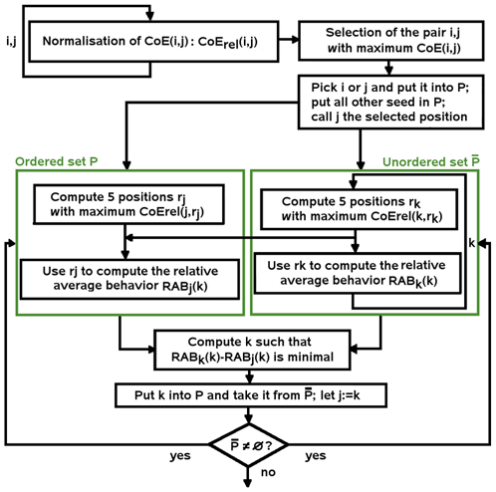
Schema of the clustering algorithm. The set of seed positions 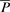 is recursively ordered into a set *P* and the iteration ends when 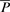 is empty. The parameter *k* in the right box associated to the unordered set 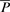, runs over all positions in 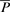. Each position 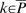 (right box) is tested with the last position *j* entering *P* (left box).

The result of the algorithm is a 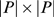 symmetric matrix indexed by seed positions ordered as *P*. Each entry of the matrix, corresponds to a relative coevolution score. The matrix can be easily represented using a color code corresponding to the interval 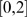, going gradually from red (high scores) to blue (low scores) passing through orange, yellow and green. One can observe large red clusters to appear in the matrix. Their boundaries are identified manually and seed positions characterizing them are claimed to be coevolved residues. A flowchart of the algorithm is given in Figure 9.

The only threshold used in the clustering algorithm is the size of a neighbouring set which is fixed to 5. This value could be parameterized but, based on the examples discussed in this paper, we evaluated the constant 5 be a good compromise between the size of coevolving networks and the minimal number of neighbouring residues necessary to estimate coevolution of two positions *i* and *j* belonging to the same network. In the ideal case, if *i* and *j* belong to the same network, their neighbours will present strong coevolution scores and the algorithm will detect the proximity of *i* and *j* by testing the differences between coevolution scores among respective neighbours. If *i* and *j* belong to a “small” coevolving network of <5 residues, then considering ≥5 positions in the neighbouring set would involve in the selection of *j* residues which do not belong to the network and noise coming from those outlier positions would disturb the selection of *j*. On the other hand, a neighbouring set which is too small in size would not provide enough information to test the stability of the coevolution signal between *i* and *j*.

The clustering algorithm has been applied to the relative coevolution score matrices of four protein families: the haemoglobin, the serine protease, the leucine dehydrogenase and PDZ domain families. The full list of residues belonging to the networks detected manually after clustering, is given in Text S1.

### Sequence alignments and distance trees

We considered 4 protein families: the haemoglobin, the serine protease, the leucine dehydrogenase and the PDZ domain families. We downloaded the sequence alignments used for the SCA analysis of the haemoglobin and the serine protease families from http://www.hhmi.swmed.edu/Labs/rr/SCA.html and used the same alignments here. The 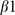 subunit of the haemoglobin family corresponds to a set of 880 aligned sequences with 161 alignment positions. The serine protease family has 616 aligned sequences with 351 alignment positions. The distance trees for these two families have been constructed from the set of aligned sequences with PHYML (using default parameters) [26].

The leucine dehydrogenase family has been analyzed with a set of 571 sequences selected by PSI-BLAST (run with the leucine dehydrogenase of *Bacillus sphaericus* as reference sequence, pdb 1LEH chain B; PSI-BLAST sequence selection parameters: E-value 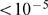 after 3 iterations). Among the 571 selected sequences, 400 display 20–30% sequence identity with the reference sequence, 140 display 40–60% and 31 more than 60%. Multiple alignment and distance tree have been realized with ClustalW (using default parameters).

The PDZ domain family has been analyzed in the same way as the leucine dehydrogenase family. A set of 1384 sequences was selected by PSI-BLAST, that was run with the third PDZ domain (PDZ3) from the synaptic protein PSD-95 of *Rattus Norvegicus* as reference sequence, pdb 1BE9 chain A. Among the 1384 selected sequences, 1263 display 20–40% sequence identity with the reference sequence, 67 display 40–60% and 53 more than 60%.

### Software availability

The program for the coevolution analysis and the clusterisation procedure can be found at http://www.ihes.fr/~carbone/data7/MaxSubTree.tgz. Relative coevolution matrices have been vizualised with a specialized viewer provided with VidaExpert and downloadable at http://www.ihes.fr/~materials. 

## Results

The combinatorial method is validated by identifying coevolved residues networks of four protein families. The haemoglobin and serine protease families have been previously analyzed in [5] using the SCA method. The leucine dehydrogenase and PDZ domain families have been analyzed using sets of sequences which were not optimized to satisfy statistical analysis constraints. While the SCA approach decided to only consider sets of sufficiently divergent sequences and detect only very clear coevolved residues explicitly excluding highly conserved residues, we preferred to work with sets of homologous sequences retrieved by PSI-BLAST search and with automatic alignment, and deal with noisy signal. Using these data, the MST method is able to detect a body of conserved positions and it is sensitive enough to meaningfully cluster such conserved positions in smaller subsets in some fine manner depending on the divergence among the sequences. This point is illustrated by the leucine dehydrogenase and the PDZ domain case studies.

For all four protein families, the method detects about 20–30% of residues as involved in networks. Here below, numbers naming residues in predicted networks refer to residue positions in the three-dimensional structure.

### Haemoglobin family

Haemoglobins are tetramers formed by two *α* subunits (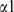, 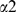) and two *β* subunits (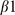, 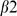), and they exist under two conformations: a T form of low affinity for oxygen and a R form of high affinity for oxygen [27]. The T form, which presents a non optimal positioning of residues in the oxygen binding site, is stabilized by an interaction network of residues at the interface between 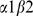 and 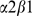 subunits [27],[28]. The binding of an oxygen molecule on one of the subunits involves a local modification of the structure which is propagated at the interface allowing a relaxation of the structure to a R form [27],[29] and the binding of oxygen molecules on the other subunits.

**Figure 10:**
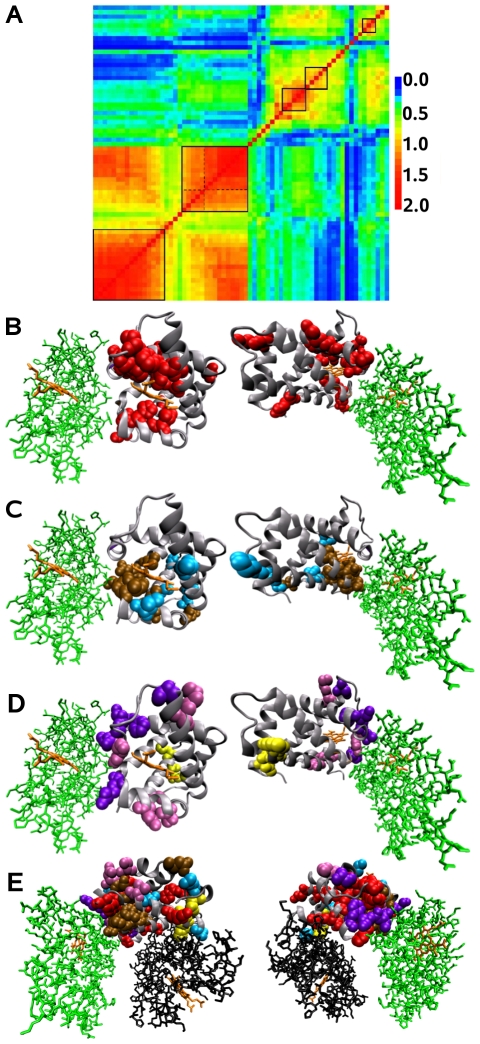
Haemoglobins. A: Matrix of relative coevolution scores 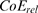. Five coevolved residues networks are detected by the MST method and manually selected (boxes limit the boundaries). Dotted lines in the second square from the bottom left distinguish two subnetworks detected by the SCA method. BCD: Coevolved residues networks in the structure of the human haemoglobin 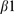 subunit (two faces of the 1HDB chain B). Residues in the networks are indicated using the Van der Walls representation, haem in orange, 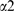 subunit in green and 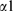 in black; B: network associated to the haem binding site (red); C: network associated to the allosteric function; residues are colored in brown and blue according to which SCA network they belong to. Brown positions are located between the haem and the 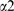 subunit binding site, and blue positions are in contact with brown positions close to the haem; D: networks associated to the 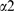 and 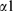 subunit binding sites; they correspond to the third (deep violet), forth (light violet) and fifth (yellow) networks in A. E: Global view of the coevolved residues networks.

Among the 161 alignment positions of the haemoglobin family, 57 (35% of aligned positions) have been selected as seed positions. Our combinatorial method applied to this family lead to the identification of five networks (Figure 10A) covering the 29% of the residues of the 1HDB chain B structure.

#### Network associated to the Haem binding site

The first network (Figure 10A, first square from the bottom left) detected for the 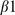 subunit, is constituted by 16 positions that are structurally closely located and that form the haem binding site where the oxygen is fixed near the 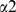 subunit interaction site (Figure 10B). Two of the most conserved positions are not linked to the others and are located behind the haem binding site. This network is not predicted by SCA since the method does not consider highly conserved positions.

#### Network associated to the allosteric function

The second network (Figure 10A, second square from the bottom left) shows two different intensities in the coevolution signal. They correspond to the two different networks detected by the SCA method applied to this family (dotted lines in Figure 10A delimit them). We detected a strong signal linking all these positions, suggesting a common evolutionary pressure, and supporting the idea that residues form one single network. The network detected by the MST method is composed of 15 positions. The first 10 positions from the bottom left in Figure 10A (97, 98, 95, 94 91, 136, 93, 84, 101 and 74) correspond to one of the two SCA networks and the remaining 5 positions (7, 119, 132, 61 and 86) correspond to the other. The second SCA network contains two more positions (112 and 118). Position 118 is alternatively mutated in serine and threonine, residues which are known to be highly interchangeable. The corresponding dispersed distribution of this residue in the distance tree forbids the detection of the position as a persistently conserved seed position. Note that position 112 is also not detected as a seed position by the SCA approach. Interestingly, position 98 detected in this network has been previously predicted to be determinant in protein interconnectivity [18].

Positions of this network are physically connected and induce a pathway between the haem binding site and the 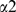 subunit interaction site, with the exception of three isolated positions (Figure 10C). Connected positions agree with the ones experimentally verified to be involved in the structural modification from the T form to the R form of the structure [29]. The close location of blue and brown positions and the fact that blue positions are not connected to each other but rather to blue ones support the idea of a unique network and justify the high coevolution scores observed for these positions.

Notice that this network presents high coevolution scores with the network associated to the Haem binding site. In fact, all positions of these two networks are very conserved and hence, their MSTs highly overlap. However, the method is able to sharply differentiate the evolution signal associated to the two different functional networks.

#### Networks associated to subunits binding sites

The third and fourth networks (Figure 10A, third and fourth squares from the bottom left) correspond to physically connected positions which are either close to or involved in the interaction site between the 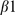 and 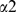 subunits (Figure 10D) and they are isolated from the haem binding site. The fifth network (Figure 10A, last square from the bottom left) corresponds to three physically connected positions which are close to the binding site between the 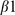 and 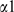 subunits (Figures 10D and 10E). These three networks are associated to the interaction sites of the chains forming the tetramer.

These three networks are not detected by SCA due to a statistical threshold intrinsic to the approach that rules them out: for this set of sequences, SCA does not consider seed positions with less than 600 sequences conserving the same residue, while all residues in these networks are conserved in at most 530 sequences.

#### A global overview of the networks

The mapping of all detected positions (Figure 10E and Text S1) provides a global view of the networks predicted for the haemoglobin family. The red positions surround the haem and seem to be involved in the binding of the haem to the 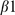 subunit. The blue and brown positions are close to the haem and 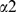 subunit bindind sites, and they seem to be involved in the allosteric function of the haemoglobin regulating the affinity of the protein for the oxygen. Violet positions are located at the 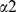 subunit binding site, far from the haem binding site. They might be used for the recognition and the binding of the two subunits. Finally, the three yellow positions, located at the opposite site of the 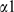 subunit binding site, might play a functional role in the interaction of the two subunits.

On this global view, all isolated positions detected in different networks are connected and they are all directly or indirectly linked to the yellow positions. All detected residues seem to form a pathway across the structure linking the 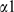 subunit binding site to the interaction residues networks associated to the haem and 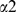 binding sites on the opposite of the structure. This observation leads to think about a functional mechanism signaling the interaction of the four chains of the haemoglobin. However such a pathway, involving interactions between residues of different networks, would require a very complex evolutionary mechanism to be conserved.

### Serine Protease Family

**Figure 11:**
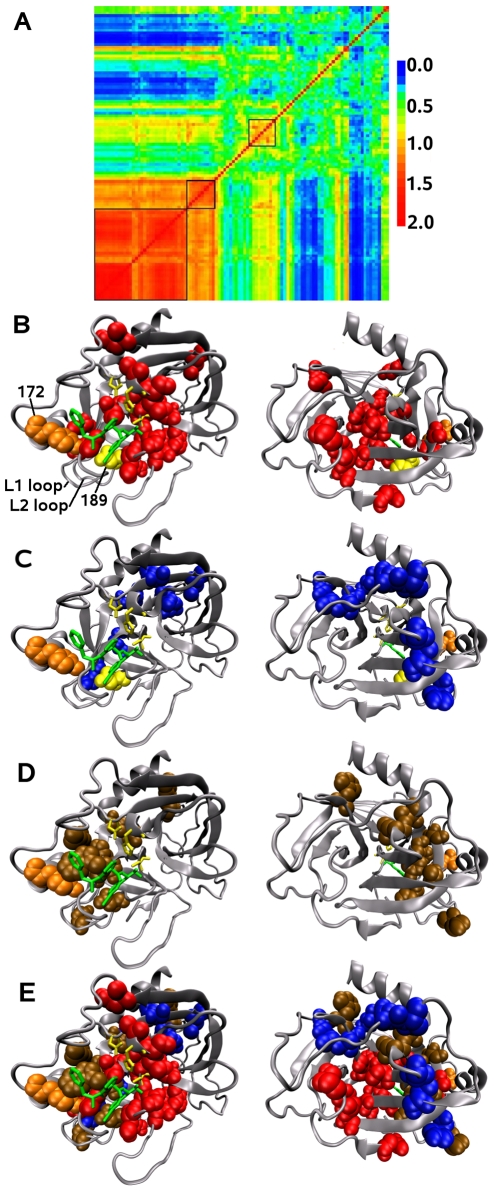
Serine proteases. A: Matrix of relative coevolution scores 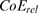 for the serine protease family. Three coevolved residues networks have been manually selected from the matrix and are indicated by black boxes. BCD: Coevolved residues network detected for the serine protease family are indicated using the Van der Walls representation in the bovine trypsin structure (two faces of the 1AUJ chain A). The catalytic triad is represented by a yellow wireframe. L1 and L2 loops supporting the S1 site are indicated. Position 172 on the L3 loop in orange and position 189 on the L1 loop in yellow are indicated using the Van der Walls representation. A substrate analog (inhibitor) of the ligand is in green; B: network associated to the catalytic site (red) except for the catalytic triad that belongs to this network; C: network with potential structural role (blue); D: network associated to the ligand specificity (brown). E: Global view of the coevolved residues networks.

Serine protease are enzymes with a catalytic triad performing the cleavage of peptidic liaison. Different serine proteases exist according to their ligand specificity. For instance, trypsins are specific to liaison involving a lysin or an arginin whereas chymotrypsins are specific to liaison involving hydrophobic or aromatic residues (preferentially phenylalanine) [30],[31]. A major determinant in the ligand specificity is the S1 pocket which interacts with the specific residue of the ligand. A negative charge (Asp189) in the bottom of the S1 pocket of trypsin suggests a local electrostatic mechanism for the specific ligand recognition of positively charged residues. However the modification of a serine protease from a trypsin to a chymotrypsin specificity requires the mutation of several positions in the S1 pocket and on the surface loops L1, L2 and L3 close to the S1 pocket [30] (indicated in Figure 11B, left). This implies that a group of residues cooperatively acts for the ligand specificity of serine proteases.

Among the 351 alignment positions of the 616 sequences of the serine protease family, MST selected 103 seed positions (29% of aligned positions). Three coevolving residues networks have been detected for this family through a manual selection (Figure 11A). These selected positions cover the 23% of the residues in the structure 1AUJ chain A.

#### Network associated to the catalytic function

The first network (Figure 11A, first square from the bottom left) corresponds to very conserved positions: 31 positions on the structure are essentially grouped around the ligand and they include the catalytic triad (Figure 11B). Most of these positions form a network of connected residues located on the S1 pocket and on the extremities of the L1 and L2 loops. Four positions are isolated and located at the opposite of the S1 pocket. This network is not predicted by SCA since the method does not consider highly conserved positions.

#### Network behind the catalytic site: a structural role

The second network is composed of 9 positions (Figure 11A, second square from the bottom left) and 7 of them agree to form the network detected by SCA (they are 184, 105, 52, 46, 201, 136, 124 and the two extra positions are 108 and 123). SCA detects one more position (81), which is not detected as seed position by MST. In fact, this position is mutated back and forth in glutamic acid and glutamin residues, that is residues which are known to be highly interchangeable. The dispersed distribution of these residues in the tree forbids the detection of the position as a persistently conserved seed position.

Most of the positions of this network are structurally close but not in contact and they are located across the structure from the L1 loop to a *β* strand behind the catalytic triad (Figure 11C). The strong connectivity in the matrix of relative coevolution scores between this network and the first one (see Figure 11A) indicates conservation of the network and hence, a potential role for the protein. The special location of the residues, their hydrophobic nature and the presence of one proline and two cysteines suggest the structural role of this network to maintain both the catalytic site and the position of the L1 loop. It was proposed that coevolved residues behind the catalytic site may make precise positioning of the catalytic residues possible [5]. The two extra positions 108 and 123 are structurally close to other positions of the network and this supports the existence of an evolutionary constraint on these two positions.

#### Network associated to ligand specificity

The third network (Figure 11A, third square from the bottom left) is composed of 9 positions among which 7 correspond to a network detected by SCA (positions 209, 215, 189, 180, 183, 228, 51, and the two supplementary positions are 186 and 231). The coevolution signal for this network is weak for both the MST and the SCA methods.

Most of the residues of this network are physically connected and located on the L1 and L2 loops supporting the S1 pocket (Figure 11D). This is in agreement with experimental observations showing the importance of the two loops in the ligand specificity. Some coevolved residues are isolated, as position 51 for instance, which is located behind the catalytic site. Position 189, crucial for ligand specificity, belongs to the network. Position 186 is not in contact with other coevolved residues but its location in the S1 pocket, in the middle of the L1 loop and close to the L2 loop, suggests a possible functional role for ligand specificity. Position 172 of the L3 loop, which has been experimentally observed to be involved in the ligand specificity [32], is not detected by the MST method and corresponds to a weak signal detected by the SCA method. This suggests that another kind of evolutionary pressure, possibly independent or conjugated to coevolution, might maintain the role of position 172 in the ligand specificity of serine protease.

#### A global overview of the networks

Coevolved positions in the three detected networks are structurally close (Figure 11E and Text S1) but essentially organized in different regions: residues that are involved in the catalytic site (red) are around the catalytic triad and on the S1 site, residues involved in the ligand specificity (brown) are mainly sitting on the S1 site located on the L1 and L2 loops, and residues involved in the structural maintaining of the functional sites (blue) surround residues belonging to the other networks from the L1 loop to the catalytic site.

Notice that positions 194 and 141 detected in the catalytic site network, position 189 detected in the ligand specificity network and position 46 detected in the network located behind the catalytic site are identified as centrally conserved positions (that is, determinant in protein interconnectivity) in [18]. Also position 172, which is not detected by MST and which presents a weak signal by SCA, is not centrally conserved but is in contact with a centrally conserved position.

### Leucine dehydrogenase family

Amino acid dehydrogenase enzymes catalyze the oxidative deamination of specific L-amino acids. Leucine and valine dehydrogenases (LeuDH and ValDH) catalyze oxidation of short aliphatic amino acids [33], glutamate dehydrogenases (GluDH) preferentially recognize glutamate [34], and phenylanine dehydrogenases (PheDH) preferentially recognize aromatic amino acids. Amino acid dehydrogenase enzymes are formed by two domains separated by a deep cleft accommodating the catalytic site. A domain supports the NAD+binding site, while the other supports the substrate binding site. Once the NAD+and the substrate are fixed, a structural modification takes place from an open to a closed conformation and locates the NAD+near to the substrate for its catalysis.

A mechanism for the basis of the differential amino acid specificity between these enzymes involves point mutations in the amino acid side-chain specificity pocket and subtle changes in the shape of this pocket caused by the differences in quaternary structure [35]. Experimental observations show that L40, A113, V291, and V294 of LeuDH are involved in the substrate specificity but different combinations of residues appear according to the enzyme specificity [36]. Positions 113 and 291 are conserved for LeuDH and GluDH but are mutated in PheDH where they play a crucial role for the substrate specificity [36]. Positions 40 and 294 are crucial for GluDH specificity but are mutated in LeuDH [37]. However, the only mutation of positions 40 and 294 in the GluDH is not sufficient to reverse the specificity of the enzyme into a LeuDH specificity and abolish its catalytic activity [37]. Besides the physico-chemical nature of the residues, a structural modification allowing for an adapted positioning of the residues in the active site is also necessary for the substrate specificity [37]. A cooperative evolution of residues involved in the structural modification from the open to the closed conformation is expected. Finally, the amino acid dehydrogenase enzymes are oligomers whose number of chains is different between the different enzymes. The complexity of the evolutionary pressures affecting the different amino acid dehydrogenases, with ligand specificity determined by a combination of constraints coming from sequence and structure, motivated us to explore this family.

**Figure 12:**
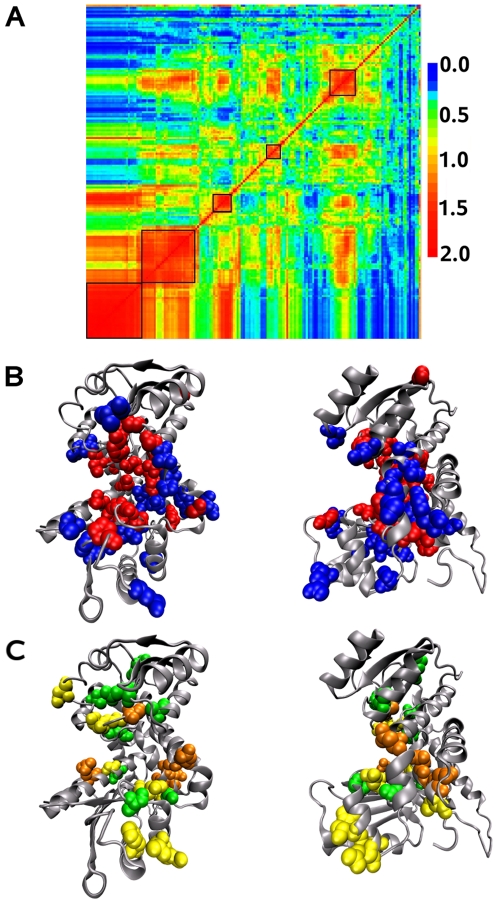
Leucine dehydrogenases. A: Matrix of relative coevolution scores 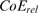 for the leucine dehydrogenase family. The 5 identified networks have been manually selected on the matrix. Signals for detection are noisy and errors in clustering positions are likely; due to red scores, the last position of the matrix, for instance, seem misplaced and better clustered with positions appeared before in the matrix. Despite the intrinsic difficulty in detection, the strong difference in signals among networks, globally justifies all five. The first and third networks display similar signals (see red scores along the associated columns and rows) but each of them shares different signals with the second network. The same is observed for the fourth and the fifth networks with respect to the third one. BC: Coevolved residues networks on the *Bacillus sphaericus* leucine dehydrogenase structure 1LEH (chain B). The catalytic site is illustrated on the front (left) and on the side (right); B: network associated to the catalytic function (red, first in A) and network associated to ligand specificity (blue, second in A); C: third (green), fourth (orange) and fifth (yellow) networks detected in A.

Among the 580 alignment positions of the 571 sequences of the amino acid dehydrogenase family, 169 (29% of the alignment positions) have been selected as seed positions. The MST method applied to this family lead to the (manual) identification of 5 networks on the relative coevolution score matrix (Figure 12A). Positions identified in the networks represent 22% of the residues in the structure 1LEH chain B. Notice that a noisy interference is observed between the different networks (this corresponds to red dots appearing in the strip just below the squares delimiting the networks).

#### Network associated to the catalytic function

The first network (Figure 12A, first square from the bottom left) detected for the LeuDH is constituted by very conserved positions. Its 28 positions form groups of physically connected residues located in the catalytic pocket. The location of these residues on the NAD+binding site (that is, the inner pocket surface of the upper domain on Figure 12B), on the substrate binding site (that is, the inner pocket surface of the lower domain on Figure 12B) and on the bottom of the catalytic pocket (where catalytic residues are located) underlines the role of these residues for the catalytic function of the enzyme. Namely, positions 80, 68 and 115 known to be involved in the catalytic function [35],[38],[39], the five glycines 41, 42, 77, 78 and 290 predicted to be involved in the shape of the active site [35], and position 150 known to play an active role in the NAD+binding site [35] are detected in this network.

#### Network associated to substrate specificity

The second network (Figure 12A, second square from the bottom left) presents several contact points with the first one and it is formed by very conserved positions. Also, these two networks overlap each other and it is hard to distinguish them, even manually. The 22 positions of the second network surround residues of the catalytic pocket. The majority of them are located under the substrate binding site and in the bottom of the catalytic pocket close to the catalytic site (Figure 12B and Text S1). No residue is observed behind the NAD+binding site, and this agrees with the idea of a substrate specificity role of this network. Positions 40 and 294 differentiating substrate specificity in LeuDH and GluDH belong to this network. Position 291, which is conserved in LeuDH and GluDH and is involved in substrate specificity for PheDH, is also detected in this network. Only position 113 involved in specificity is detected in the first network. Notice that it has been reported that, in LeuDH, important determinants of the differential substrate specificity come not only from the substitutions of Lys89 and Ser380 in GluDH by Leu40 and Val294 in LeuDH to change the chemical nature of the substrate binding pocket, but also from the subtle changes in the pocket shape that arise from the difference in quaternary structure [35].

Isolated positions are also observed but their location at the periphery of the catalytic site suggests a structural role possibly required to create contacts in the closed conformation.

The overlap and the several contact points between the first and second network as well as the presence of position 113 in the first network and the noise observed on the relative coevolution score matrix, suggest that more sequence divergence would be necessary to make the signal clearer. Despite this lack of divergence among sequences, it is interesting to observe that the MST approach identifies and distinguishes pertinent functional positions which are known to be involved in the catalytic function and in substrate specificity.

#### Other networks

Three other “networks” are identified in the relative coevolution score matrix and they are composed of residues which turns out to be sparsely located on the structure, hence not showing a coherent behavior explainable by functional purposes (Figure 12C). These networks might be due to a phylogenetic signal rather than to a functional coevolution signal. The noise associated to the networks (characterized by red signals lying on the columns and rows of the positions defining the network) is most likely due to insufficient sequence divergence and it may be used to discriminate phylogenetic from functional signals. Indeed the idea is that the “noise” associated to the networks could be used to evaluate the accuracy of the observed networks on the relative coevolution score matrix and be exploited for an automatic selection of pertinent networks. This will be done elsewhere.

Notice that amino acid enzymes are oligomers and that no interaction site is detected by the method. The fact that not all amino acid dehydrogenases share the same number of monomers and interacting sites can explain the absence of a signal.

### PDZ Domain Family

PDZ domains are small globular interaction modules whose function is to mediate protein-protein interaction by binding to the C-terminus of the target protein in a sequence-specific fashion. PDZ domains are often found in combination with other interaction modules and they play diverse role in cells such as in organizing diverse cell signaling assemblies, in establishing cell polarity, in directing protein trafficking and in coordinating synaptic signaling [40]–[42].

The PDZ domain family is divided into distinct classes on the basis of target sequence specificity: class I domains bind to peptide ligands of the form -SER/THR-X-VAL/ILE-COO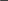, and class II domains bind to sequences of the form -PHE/TYR-X-VAL/ALA-COO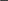 [43],[44]. In class I PDZ family, the key residue responsible for ligand specificity is H372 [45] and it forms hydrogen bonds with the Ser or Thr hydroxyl group of the ligand recognition motif [46]. From covariance data, H372 appears to be coupled strongly to F325 located within the core of the protein, and to position L353 on the opposite site of the binding pocket. Together, these residues map out a potential signaling pathway whose functional importance has been largely confirmed by experimental mutagenesis.

**Figure 13:**
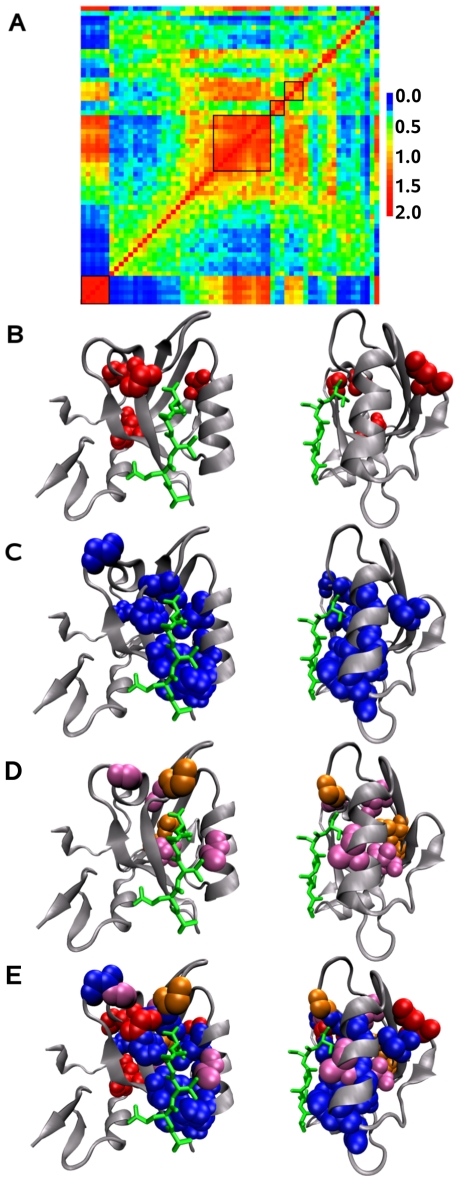
PDZ domains. A: Matrix of relative coevolution scores 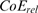 for the PDZ domain family. The 4 identified networks have been manually selected on the matrix. Signals for detection are noisy and errors in clustering positions are likely; due to red scores, the last position of the matrix, for instance, seem misplaced and better clustered with positions appeared before in the matrix. BCD: Coevolved residues networks on the third PDZ domain of PSD-95 1BE9 (chain A). The binding site is illustrated on the front (left) and on the side (right) bound to a peptide indicated in green. Residues in the networks are indicated using the Van der Walls representation. B: network associated to highly conserved structural positions (red, first in A); C: network associated to peptide specificity (blue, second in A.); D: third (orange, third in A) and fourth (violet, fourth in A) networks surrounding the network associated to ligand specificity. E: Global overview of the four networks.

Among the 186 alignment positions of the 1384 sequences of the amino acid PDZ domain family, 63 (34% of the alignment positions) have been selected as seed positions. The MST method applied to this family leads to the (manual) identification of four networks on the relative coevolution score matrix (Figure 13A). Positions identified in the networks represent 21% of the residues in the structure 1BE9 chain A.

The position numbering used here follows the reference structure 1BE9, the corresponding numbering used in ATD and SCA is His76 (position 372), A80 (position 376), K84 (position 380), G33 (position 329), G34 (position 330), F29 (position 325), G26 (position 322), A51 (position 347), L57 (position 353), V66 (position 362), V90 (position 386), I31 (position 327) and I45 (position 341).

#### Network associated to conserved positions

The first network (Figure 13A, first square from the bottom left) detected for the PDZ domain is constituted by 6 very conserved positions (324, 356, 357, 363, 347 and 351). These positions are not physically connected to each other and appear sparsely located on the structure (Figure 13B). Contrary to haemoglobin which recognizes a specific molecule (haem) and to enzymes exhibiting a specific catalytic site, both requiring strong residue conservation, no conserved residues are observed in the proximity of the binding site for the PDZ domain. This might be expected for proteins that recognize different sequence specific binding sites. Notice that most positions detected in this network are key structural positions located at the extremities of regular secondary structures initiating variations in the direction of the backbone.

#### Network associated to ligand specificity

This network (Figure 13A, second square from the bottom left) is composed of 12 positions. Ligand specificity networks have been predicted by SCA (statistical) and ATD (molecular dynamics) which share four common positions. Except for position 347 which is found in our conserved network, all other three positions (372, 325 and 353) are common to our prediction for the ligand specificity network. Together, these three residues map out a potential signaling pathway whose functional importance has been largely confirmed by experimental mutagenesis. Differences also appeared between the ATD and SCA approaches. Among the two positions which are found by ATD but not by SCA (327 and 341), position 327 belongs to our network. Among the seven positions which are found by SCA but not by ATD (376, 380, 329, 330, 322, 362 and 386), positions 329, 330 and 362 belongs to our network. Finally, positions 336, 323, 379, 344 and 375 are found in our network but not by SCA nor ATD. Notice that position 379 has been previously predicted to be determinant in protein interconnectivity in [18] as well as position 325 above. Except for positions 344 and 353 which are isolated on the structure, all other positions detected by MST are physically connected and form the binding site (Figure 13C).

#### Other networks

Residues detected in the two other networks (Figure 13A, third and forth squares from the bottom left) are not physically connected together but are connected to residues involved in the ligand specificity network (Figure 13D and 13E). The third network is composed of 3 positions (322, 388 and 390) and the fourth one by four positions (376, 359, 316 and 345). Positions 322 and 376, which are detected in the two networks, are detected by SCA in the ligand specificity network but not by ATD. Their MST detection in other than the ligand specificity network and their absence in ATD prediction suggest that these positions might be not directly involved in ligand specificity as proposed in [5]. However their high conservation and their location around the ligand specificity network hint for another role they might play within the PDZ domain, maybe structural for maintaining the binding site.

Notice that mutations on positions 362 found in the ligand specificity networks predicted by SCA and MST, 386 found in the ligand specificity network predicted by SCA only, and 316 found in the fourth MST network have been shown to have effect on the rate constant for the PDZ2-peptide binding reaction (where PDZ2 is the second PDZ domain of the protein) but little effect for the PDZ3-peptide binding reaction [47]. Thus the detection of these positions by approaches like SCA and MST which use evolutionary signals, can be due either to a reminiscent evolutionary signal present in homologous sequences or to a weakening of the functional role in the binding.

## Discussion

### Conservation and coevolution

The notions of conservation and mutual conservation might appear at first to be distinguished concepts but our combinatorial approach exploits the idea that along time evolution, conservation comes before coevolution and that conservation occupies a specific position within the continuum spectrum where to measure different degrees of coevolution. The intended model that we use identifies a protein sequence as an object that evolves through mutations which are driven by the potential key functional or structural role of the positions. If two or more residues cooperate, they will coevolve together. Depending on the evolutionary constraints due to folding, maintenance of allosteric properties, degree of specificity of the interaction with other molecules, signals of coevolution will be more or less strong. Notice that two positions which are fully conserved are treated by the method as “perfectly coevolving” (in this sense, conservation can be mathematically treated as an extreme case of coevolution).

The serine protease family is a reference example, discussed here and in [48], that underlies the idea of “continuity” in proteic sequence evolution. Residues involved in protein folding, catalytic triad and ligand specificity are conserved within sequences of the trypsin and chymotrypsin families but their degree of conservation is different depending on their role. Residues involved in protein folding and catalytic triad are essentially the same for all serine protease. Residues involved in ligand specificity have a strong family specificity, resulting in two different sets of residues distinguishing trypsin from chymotrypsin. These latter are driven by different evolutionary pressures and can be revealed by a coevolution analysis.

The sharply separated signal on the relative coevolution score matrix that makes easy network detection for the serine protease family, reflects the strong sequence divergence of this family. In contrast, the leucine dehydrogenase family, which displays a moderate sequence divergence, exhibits the “overlapping” of two networks of very well conserved residues within the relative coevolution score matrix. This overlapping seems to support the idea of “continuity” of the evolutionary process transforming conserved residues into coevolved ones. In fact, despite strong residue conservation, the signal allows to distinguish the network associated to the catalytic site from the substrate specific one.

In the MST based combinatorial model, the distance tree organizing the pool of existing homologous sequences traces the evolutionary process. The distinction between conserved and coevolved positions depend on the number of MSTs associated to the position. Conserved positions are associated to few (in the ideal case just one) MSTs and they turn out to have a high score of coevolution with all other conserved positions due to a strong overlapping of MSTs. This means that if we try to match the MSTs of a conserved position against a random combination of few MSTs covering the same tree, the expected coevolution score is high. In this sense, the high score of coevolution for conserved positions is representative of an independent evolutionary pressure. The notion of “few” above depends on the family of sequences that we look at. Very divergent sequences will associate many trees to most positions and little divergent sequences will associate few trees to most positions.

### Networks detection and variable sequence divergence

A thorough analysis of correlated changes of amino acids becomes of crucial relevance for the understanding of biological functions and mechanical properties of proteins. A high sequence divergence and an appropriate size of the alignment appeared to be critical in obtaining statistical significant correlations between residues in a protein family [5],[13]. These constraints limit the analysis of well represented protein families. For instance, data used here for the haemoglobin and the serine protease families analysis were optimized in terms of sequence divergence and alignment size [5] for a statistical analysis with SCA. The MST approach used on this data was able to detect a very clear signal of coevolution for interacting amino acids involved in the function of the proteins and has detected all networks previously revealed by SCA. However, MST has allowed the analysis of a larger number of seed positions compared to SCA which is limited by statistical constraints and new networks of functional interest have been identified. Three small networks of connected amino acids located on the structure close to the interaction sites have been detected by MST for the haemoglobin family, while no other network has been detected for the serine protease family, compared to what has been found by SCA already.

For the leucine dehydrogenase and the PDZ domain family, the set of sequences and the alignments used for the analysis have not been optimized (it results from a simple PSI-BLAST detection and alignment with ClustalW). These sequences might be limited in number and their divergence might be not very high. We demonstrated that the lack of high divergence of these sequences, compared to the set of sequences used by SCA, still allows MST to identify networks associated to positions known to be involved in protein function and therefore to catch a pertinent coevolution signal. Constraints on sequence divergence imposed by statistical approaches like SCA do not allow for the analysis of the leucine dehydrogenase family even though insights into functional mechanisms are actually revealed by MST. Also, good agreement obtained for the PDZ domain family with different approaches like SCA (statistical) and ATD (molecular dynamics), and with centrally conserved positions (structural information) supports the pertinency of the networks detected by MST using sequences of variable divergence.

In general, it is difficult to evaluate predictions of a method by referring to another prediction method. The experimental predictions of highly correlated residues reported in the literature allowed to validate residues detected by MST, SCA and ATD. However experimental results on coevolved residues are few and new ones would be highly desired. Evaluations obtained by comparing different methodologies, like SCA, ATD, and detection of centrally conserved positions, on several protein structures, show that these methods do not fully agree in their predictions, and novel hypothesis on non-tested detected positions might inspire new biological experiments. We see this as an important point for these methods to be appreciated.

In conclusion, a high sequence divergence appears to be necessary for a fine analysis and a more accurate selection of functional networks. Under these conditions, MST detects pertinent signals of coevolution. In general, MST can detect a larger set of potential positions compared to statistical approaches like SCA since the constraint on sequence divergence is dropped. Under moderate sequence divergence, a noisy signal might be observed but pertinent functional information may be revealed anyway. In this sense, MST becomes a tool for biologists to detect a number of potentially functional and structural positions in protein families based on possibly loose conditions that are satisfied by the set of homologous sequences. This enlarges the spectrum of applicability of MST compared to approaches like SCA.

### On the mathematics used to study coevolution

The method introduced in this paper provides a mathematical framework where the concept of coevolution can be “structured”. The combinatorial notions help to bring out the interaction between coevolving information within the subtrees of a tree of sequences. At first sight, the method might look complicate, as often do combinatorial approaches, but the advantage, compared to more implicit approaches of algebraic or statistical nature, is that combinatorial methods are based on a direct understanding of the building blocks involved in a construction. On the contrary, implicit approaches bring little intuition on these building blocks. A main purpose for future investigation is to highlight different signals of coevolution within a protein family by suggesting formal properties that will distinguish different groups of coevolving “motifs” within a protein sequence. Some of these properties might be of structural nature and correspond to non obvious overlapping of coevolving motifs. This kind of “structures” organising groups of coevolving residues are not studied by available approaches. We expect combinatorics to help to bring new insights into evolutionary signals in protein sequences.

## Supporting Information

Figure S1 Comparison between ranks based on different definitions of a gap for the hemoglobin family. Rank distributions where gaps are considered to be different residues (red) or the same residue (green) for positions with R(T,s)>500 computed for the set of aligned sequences and associated distance tree of the hemoglobin family.
(0.09 MB TIF)

Text S1 Amino acids positions in detected networks for the four protein families analyzed in the paper.
(0.02 MB PDF)
