# Supplementary figures and images for: A Combinatorial Approach to Detect Coevolved Amino Acid Networks in Protein Families of Variable Divergence
Source: PLoS Comput Biol. 2009 Sep 4;5(9):e1000488. doi: 10.1371/journal.pcbi.1000488 (PMC2723916; doi:10.1371/journal.pcbi.1000488)

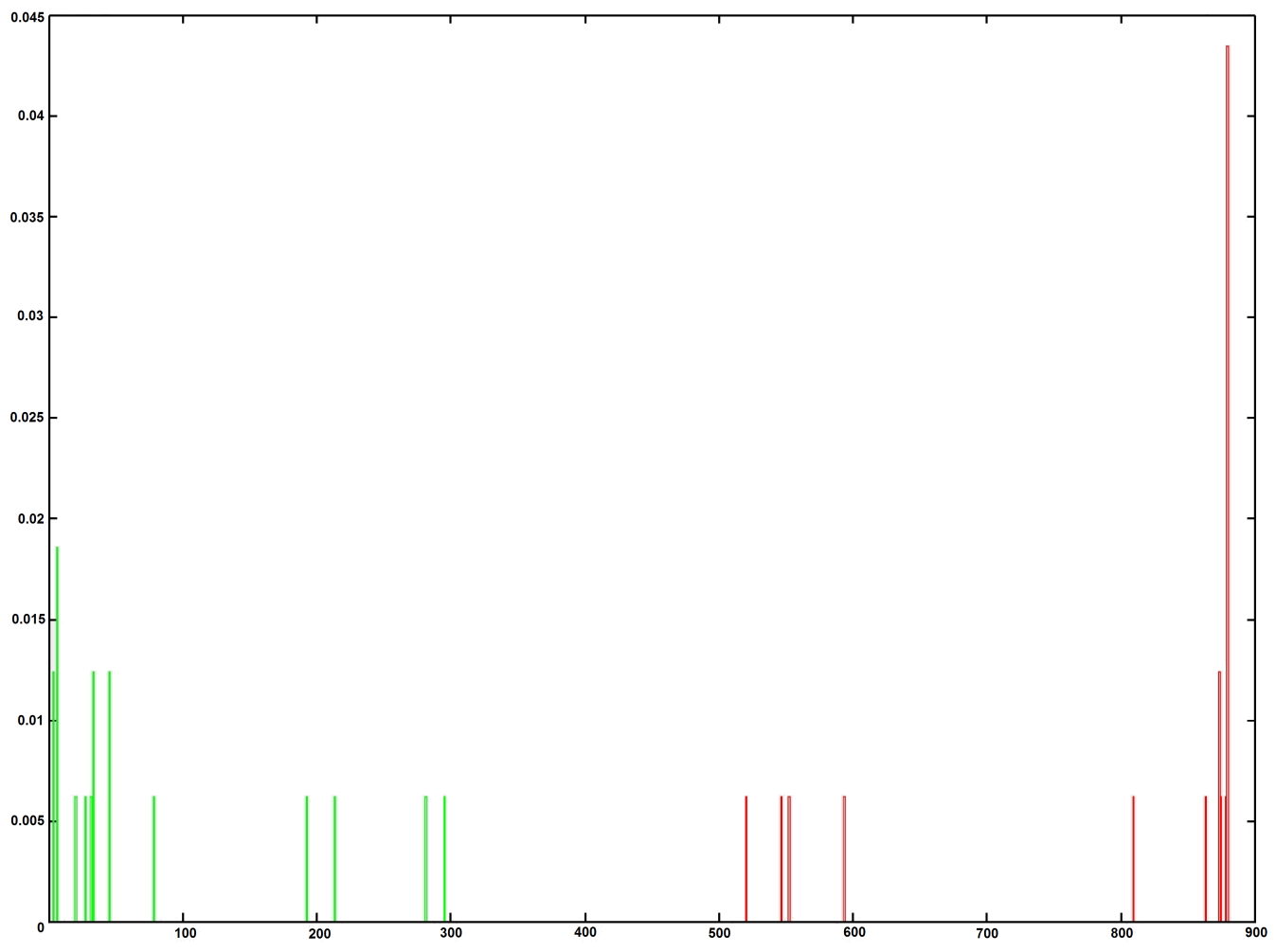

Supplement: Figure S1 — Comparison between ranks based on different definitions of a gap for the hemoglobin family. Rank distributions where gaps are considered to be different residues (red) or the same residue (green) for positions with R(T,s)>500 computed for the set of aligned sequences and associated distance tree of the hemoglobin family. (0.09 MB TIF) [file pcbi.1000488.s001.tif]
