# Supplementary material for: A Combinatorial Approach to Detect Coevolved Amino Acid Networks in Protein Families of Variable Divergence
Source: PLoS Comput Biol. 2009 Sep 4;5(9):e1000488. doi: 10.1371/journal.pcbi.1000488 (PMC2723916; doi:10.1371/journal.pcbi.1000488)
Supplement: Text S1 — Amino acids positions in detected networks for the four protein families analyzed in the paper. (0.02 MB PDF) [file pcbi.1000488.s002.pdf]

Hemoglobin family (reference structure 1HDB, chain B)

Network associated to the haem binding site (red) : 83, 59, 87, 43, 37, 58, 127, 140, 39, 25, 29, 31, 62, 16, 46, 14
Network associated to the allosteric function :
- brown : 95, 91, 98, 84, 93, 94, 97, 101, 74, 136
- blue : 132, 61, 86, 7, 119

New networks associated to the protein-protein complex interfaces :
- deep violet : 33, 52, 40, 92, 41
- light violet : 135, 42,96, 85, 81
- yellow : 124, 6, 122

Serine protease (reference structure 1AUJ, chain A)

Network associated to the catalytic function (red) : 196, 198, 194, 182, 108, 140, 142, 102, 58, 57, 55, 68, 141, 216, 214, 155, 91, 28, 43, 44, 19, 42, 211, 225, 195, 197, 193, 191, 56, 18
Network located behind the S1 site (blue) : 184, 105, 108, 52, 46, 201, 136, 124, 123
Network associated to the ligand specificty (brown) : 209, 215, 189, 180, 186, 183, 228, 51, 231

PDZ domain (reference structure 1BE9, chain A)

Network associated to highly conserved positions (red) : 324, 356, 357, 363, 347, 351
Network associated to ligand specificity : 336, 327, 323, 379, 362, 353, 372, 325, 344, 375, 330, 329
Other networks :
- orange : 322, 388, 390
- violet : 376, 359, 316, 345

Leucine dehydrogenase (reference structure 1LEH, chain B)

Network associated to the catalytic function (red) : 77, 180, 182, 78, 282, 80, 115, 262, 68, 260, 289, 290, 236, 196, 41, 42, 61, 153, 150, 238, 113, 287, 38, 37, 335, 297, 87, 141
Network associated to the ligand specificty (blue) : 75, 142, 301, 204, 93, 149, 291, 183, 47, 81, 40, 29, 112, 294, 146, 85, 110, 264, 267, 151, 130, 27
Other networks :
- green : 135, 273, 118, 179, 185, 66, 283, 232
- orange : 295, 145, 293, 296, 60, 237
- yellow : 245, 298, 125, 128, 114, 100, 263, 240, 64, 96
